# Prophylactic cranial irradiation for small cell lung cancer in the era of immunotherapy and molecular subtypes

**DOI:** 10.1097/CCO.0000000000001111

**Published:** 2024-12-02

**Authors:** Veronika Pozonec, Maria Dorothea Pozonec, Clemens Aigner, Joachim Widder, Kristiina Boettiger, Zsolt Megyesfalvi, Balazs Dome

**Affiliations:** a Department of Thoracic Surgery, Semmelweis University and National Institute of Oncology; b Multidisciplinary Centre of Head and Neck Tumors, National Institute of Oncology; c National Koranyi Institute of Pulmonology, Budapest, Hungary; d Department of Thoracic Surgery; e Department of Radiation Oncology, Comprehensive Cancer Center, Medical University of Vienna, Vienna, Austria; f Department of Translational Medicine, Lund University, Lund, Sweden

**Keywords:** immunotherapy, prophylactic cranial irradiation, small cell lung cancer

## Abstract

**Purpose of review:**

Small cell lung cancer (SCLC) is an aggressive disease with a poor prognosis, whereas its metastatic capacity carries a predilection for the brain. Although prophylactic cranial irradiation (PCI) has been used to address this problem, upcoming alternatives might necessitate reflection of its application in SCLC treatment.

**Recent findings:**

The addition of immunotherapy to treatment guidelines has provided a new strategy for the management of brain metastases. Complementation of immunotherapy with active MRI surveillance could potentially replace PCI and avoid irradiation-related cognitive side effects. SCLC's molecular profile is heterogeneous, with differential response to treatment modalities between subgroups. Investigation of these variances might be essential to improve therapeutic outcomes in SCLC patients.

**Summary:**

The role of PCI in SCLC treatment must be examined in light of immunotherapy. We summarize recent results, bearing SCLC subtypes and therapeutic vulnerabilities in mind, to derive tailored treatment strategies for SCLC patients in future settings.

## INTRODUCTION

Small cell lung cancer (SCLC) is an aggressive disease that makes up only 13–15% of all lung cancer cases worldwide. Its poor prognosis is reflected in dismal outcomes, with 5-year overall survival (OS) rates of less than 7% [1^▪▪^]. In the majority of cases, the tumor has already disseminated outside the chest by the time of diagnosis, preventing surgical resection as a therpeutic option. Patients diagnosed with extensive-stage disease (ES) are treated with systemic therapy comprising combinations of chemotherapy (CHT) and/or immunotherapy with local radiotherapy reserved for mediastinal consolidation after good partial response to systemic therapy alone [2]. Although the most common sites of distant organ metastasis include the brain, bones, liver, and adrenal glands, progression is most frequently seen in the thorax and brain [2,3]. Bulky primary tumors are often the reason for thoracic treatment failures. Furthermore, the brain is considered a ‘sanctuary site’ for tumors because of the blood–brain barrier [4^▪^]. As CHT cannot cross this barrier, prophylactic cranial irradiation (PCI) was introduced as a standard treatment strategy to improve OS for limited-stage disease (LS-SCLC) after complete remission to thoracic radiotherapy combined with CHT [5–7].

Nearly all SCLCs exhibit biallelic inactivation of the tumor suppressor genes *TP53* and *RB1*[8]. Approximately 94% of patients are ever-smokers and SCLC tumorigenesis is strongly associated with tobacco consumption [1^▪▪^]. Consequently, patients carry a high tumor mutational burden (TMB), which perpetuates disease progression. Interestingly, increased TMB has been correlated with positive responses to immunotherapy, underlining potential benefits of immunotherapy in SCLC treatment [9].

Although SCLC is still treated as a homogeneous disease in clinical settings, recent advancements have aimed to classify the molecular profile into four distinct subgroups according to the gene expression of relevant transcription factors or immune system characteristics [10]. SCLC-A and SCLC-N, defined by higher expression of the transcription factors ASCL1 and NEUROD1, respectively, reflect neuroendocrine subgroups through higher expression of neuroendocrine markers (e.g. synaptophysin or CD56), whereas POU2F3-expressing SCLCs (SCLC-P) constitute a variant, non-neuroendocrine phenotype [11]. The fourth subgroup, SCLC-I exhibits inflamed gene signatures, mesenchymal features, and low levels of the transcription factors ASCL1, NEUROD1, or POU2F3 [1^▪▪^]. Importantly, these subgroups have been associated with varying responses to therapy [12^▪^].

Here, we offer an overview of recent advancements in SCLC treatment in light of molecular subtyping efforts and pay special attention to the role of PCI. 

**Box 1:**
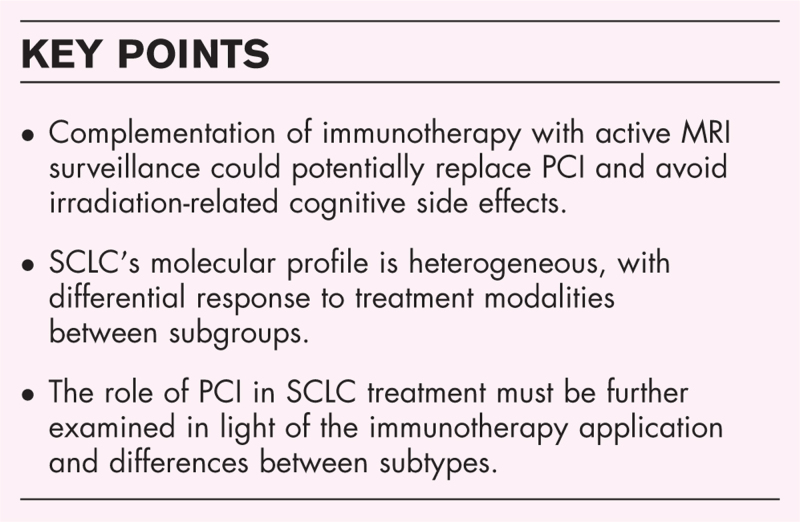
no caption available

## CURRENT THERAPEUTIC APPROACHES IN SMALL-CELL LUNG CANCER

Approximately 60–65% of patients present with metastatic spread outside the chest and are, therefore, classified as ES-SCLC patients upon diagnosis [13,14]. Furthermore, select results from clinical trials on the application of surgery have underlined that surgical resection is often not a viable therapeutic option, even in earlier stages [15,16]. According to the National Comprehensive Cancer Center Guidelines, surgical resection is only recommended in LS-SCLC (I-IIA), equating to roughly 5% of SCLC patients [17^▪▪^]. For these patients with very LS-SCLC (T_1_-T_2_N_0_M_0_), three-year survival rates of more than 50% have been achieved after undergoing definitive lobectomy and mediastinal lymph node dissection followed by postoperative systemic therapy [18,19]. In case of mediastinal lymph node metastases (N_1_–N_2_) or residual disease (R_1_ or R_2_) after surgical resection, mediastinal radiotherapy is advised to decrease local disease recurrence rates [17^▪▪^,^20^].

The benefit of PCI in stage I patients who have undergone definitive therapy and have a lower risk of developing brain metastases remains unclear [21]. Radiotherapy can be potentially applied in all stages as part of definitive or palliative therapy [17^▪▪^]. For inoperable patients with LS-SCLC (T_1_–T_2_N_0_M_0_), stereotactic body radiation therapy (SBRT) of the primary tumor followed by adjuvant systemic therapy could be beneficial [17^▪▪^]. Furthermore, mediastinal radiotherapy is also recommended in specific postoperative cases, especially in unexpected N2, considering that upfront N2-stages should not undergo surgery. A phase III randomized trial demonstrated that consolidative mediastinal irradiation (10 × 3 Gy) significantly improves 2-year OS rates and 6-month progression-free survival (PFS) in ES patients with clinical response to previously administered CHT [22]. The phase II/III RAPTOR trial (NCT04402788) is currently testing the addition of radiotherapy to the immunotherapeutic agent atezolizumab (anti-PD-L1) in ES-SCLC. Brain dissemination is conventionally treated with whole brain radiation therapy (WBRT, 10 × 3 Gy or shorter schedules). However, selected patients in good condition with only a few brain metastases might benefit from stereotactic radiotherapy [23].

Systemic CHT is the main treatment modality in SCLC management, with a prominent role in all stages of SCLC [17^▪▪^]. Etoposide in combination with platinum-based agents such as cisplatin or carboplatin (EP) has been the standard-of-care since the 1980s [24]. Despite higher response rates in the beginning of treatment, many patients relapse within the first year and portray a median OS of approximately ten months [25–27]. Notably, recent studies showed that the addition of immunotherapy to the therapeutic armamentarium prolonged median OS rates by 2–4 months in patients with ES disease [28^▪^,^29^]. Based on the promising results of the double-blinded randomized phase III IMpower133 study from 2019, the US Food and Drug Administration (FDA) and the European Medicines Agency approved the use of atezolizumab for first-line treatment of ES-SCLC in combination with EP [29,30]. Durvalumab, another immunotherapy agent targeting programmed death ligand 1 (PD-L1), has also been approved based on data from the randomized phase III CASPIAN trial in ES settings [31,32]. Durvalumab has also significantly increased OS in LS-SCLC according to recent results of the ADRIATIC phase III trial [33^▪^]. The guidelines for subsequent systemic treatment options are not as clearly established and depend on previously administered therapeutic agents as well as the length of the disease-free interval [1^▪▪^]. Among others, second- and further-line treatment options include topotecan, irinotecan, lurbinectedin, tarlatamab, temozolomide, cyclophosphamide, nivolumab, pembrolizumab, gemcitabine, paclitaxel, or docetaxel [17^▪▪^].

## PROPHYLACTIC CRANIAL IRRADIATION IN THE ERA OF IO

SCLC is notorious for its tendency to disseminate and develop metastases in the brain [9,34^▪^,^35^]. Additionally, the presence of brain metastases denotes poor prognosis [35]. More than two-thirds of patients are detected when brain metastases are already present, and the risk of devloping brain metastases after CHT remains at around 50% due to poor drug permeability through the blood–brain barrier [34^▪^,^36^]. Hence, PCI was implemented to decrease the occurrence of brain metastases and improve survival [9,34^▪^]. Today's indication for PCI is based on a meta-analysis of seven trials conducted by Aupérin's working group. Researchers analyzed data from nearly 1000 patients in all SCLC stages who had a complete response (CR) to chemoradiotherapy (CRT) (>75%) or CHT alone [5]. Results demonstrated an overall reduced incidence of brain metastases and improved OS. However, factors such as the assessment of CR solely by thoracic X-rays and the inclusion of select trials that had been performed prior to the MRI era most likely influenced study outcomes. Numerous other trials with positive outcomes have followed thereafter (Table 1). Despite favorable results in these trials, many enrolled patients lacked a baseline MRI scan [37^▪^] and medical imaging has experienced major improvements since then. Especially higher imaging resolution and more frequent use of cranial MRI for both SCLC staging and follow-up has generated interest to offer screening MRI at follow-up for neurologically asymptomatic patients.

**Table 1:** Clinical trials on prophylactic cranial irradiation

Trial	Phase	Date	Enrolled	SCLC stage	Experimental arm	Control	Outcomes
NCT00016211	3	2001–2006	287	Extensive	PCI	Observation	BM incidence 40% control vs. 15% PCI PCI longer DFS, OS 1-year survival rate 27% PCI group vs. 13% control Acute and late toxicity acceptable
NCT00005062	3	1999–2005	720	Limited	High-dose PCI over 16 or 24 days	Standard-dose PCI over 10 days	Two-year follow-up: no significant difference in BM incidence OS 42% standard-dose, 37% higher dose Five serious adverse events in standard-dose group vs. zero in the higher-dose group
NCT00057746	2	2009–2013	265	Limited	36 Gy PCI – 2.0 Gy once daily, 18 fractions – 1.5 Gy twice daily, 24 fractions	25 Gy PCI - 2.5 Gy once daily, 10 fractions	No significant differences in QoL and Hopkins Verbal Learning Test 1 year later increase in CNt in the 36-Gy cohort
NCT00006349	3	2001–2007	9	–	oral donepezil daily and vitamin E + PCI	Oral placebos + PCI	*Only nine enrolled, no definitive conclusions*
NCT00006344	3	2000.05–2000.12	0	Limited	Radiotherapy to the left cerebral hemisphere after WBRT	Radiotherapy to the right cerebral hemisphere after WBRT	*Terminated*
NCT01055197	2	2010–2016	97	Extensive	PCI + cRT	PCI	*At planned interim analysis, the study crossed the futility boundary for OS and was closed* 1-year OS no difference 3- and 12-month progression rates 53.3 and 79.6% for PCI vs 14.5% and 75% for PCI+cRT
TULIP NCT01486459	NA	2011–2014	7	–	Lithium + PCI	PCI	*Insufficient recruits*
NCT01553916	1/2	2012–2017	19	–	Lithium carbonate + PCI	PCI	*No study results posted*
NCT01780675	3	2013–2018	168	–	HA-PCI	PCI	No significant differences between the two
NCT01797159	2	2013–2019	20	Limited	HA-PCI	historical control (RTOG 0212)	Two-year OS 88% no significant decline in performance MRI revealed asymptomatic brain metastases in 20% Two patients developed metastasis in the under-dosed region
HIPPO-SPARE 01 NCT01849484	2	2013–2021	35	–	HA-PCI	PCI	Complex pathophysiological changes in cerebral microstructures after radiation Hippocampal microstructure differed (HA-PCI vs. PCI) after 6 months
SAKK 15/12 NCT02058056	2	2014–2017	44	Limited	HA-PCI	–	6 months: 34.2% patients no NCF decline 12 months: BMFS 84.2% and OS 87.7%
NCT02366741	Pilot	2015–2017	5	Limited	HA-PCI	–	*Unknown status, no study results posted*
PREMER-TRIAL NCT02397733	3	2014–2020	150	–	HA-PCI	PCI	DFR decline HA-PCI (5.8%) vs. PCI (23.5%) DFR (11.1 vs. 33.3%), total recall (20.3 vs. 38.9%) total free recall (14.8 vs. 31.5%) BM incidence, OS, and QoL were not significantly different
NCT02605811	2	2015–2021	426	Limited	Temozolomide	PCI	*Unknown status, no study results posted*
NCT02635009	2/3	2015–(2027)	418	–	HA-PCI	PCI	*Active, not recruiting*
NCT02736916	NA	2016–2018	3	Limited	HS-WBRT PCI	PCI	*Unknown status, no study results posted*
NCT02906384	2	2016–2020	154	–	HA-PCI	PCI	*Unknown status, no study results posted*
NCT03514849	NA	2018–(2026)	(360)	–	PCI	Placebo	*Recruiting*
S1827 (MAVERICK) NCT04155034	3	2020–(2027)	(668)	–	MRI Active Surveillance	PCI + MRI surveillance	*Recruiting*
NCT04535739	3	2019–2022	414	Extensive	PCI	Observation	*Unknown status, no study results posted*
PRIMALung Study NCT04790253	3	2022–(2028)	(600)	–	MRI Active Surveillance	PCI + MRI surveillance	*Recruiting*
NCT04829708	3	2021–(2028)	(534)	Limited	MRI Active Surveillance	PCI + MRI surveillance	*Recruiting*
NCT04947774	NA	2020–2022	100	Extensive	PCI	Observation	*Unknown status, no study results posted*
NCT05651802	NA	2023–(2026)	(220)	Limited	MRI Active Surveillance	PCI + MRI surveillance	*Recruiting*

BM, brain metastasis; BMFS, brain metastasis-free survival; CN, chronic neurotoxicity; cRT, consolidative extracranial radiotherapy; DFR, delayed free recall; DSF, disease-free survival; HA, hippocampus avoidal; NCF, neurocognitive function; OS, overall survival; PCI, prophylactic cranial irradiation; QoL, quality of life; WBRT, whole brain radiotherapy.

In contrast to conventional CHT immunotherapy agents are able to penetrate the blood–brain barrier. Introduction of immunotherapy to SCLC treatment regimens has supported the idea (or possibility) of omitting PCI from therapeutic regimens, but there is currently insufficient data on immunotherapy efficacy to prevent brain metastases from SCLC to provide an answer [34^▪^,^38^]. Although the Impower-133 study permitted PCI inclusion [29], only 11% of the study population (*n* = 22/arm) received this treatment modality. Results from both the whole cohort and the posthoc subgroup analysis without PCI treatment showed no difference. This might suggest that immunotherapy alone was effective in delaying or preventing brain metastases and the beneficial effect was not dependent on PCI application. However, this observation needs further validation [39^▪^]. In contrast, the CASPIAN trial excluded PCI from the experimental arm, although PCI was permissible for patients in the control group [31]. A subsequent analysis of this study reported significantly increased time for brain metastasis formation in the durvalumab-CHT arm as opposed to the control group that received PCI [40].

PCI has many side effects including neurocognitive toxicity [9,34^▪^,^35^,^41^,^42^]. Even lower radiation doses can contribute to a significantly worse quality of life, memory loss, or decreased neurocognitive functions [9]. Recently, hippocampal-avoidance PCI (HA-PCI) has been suggested and is supported by NCCN guildeines [17^▪▪^,34^▪^,37^▪^]. Although HA-PCI efficacy is similar to PCI, studies have shown considerable delay in or reduction of the severity of cognitive deterioration [1^▪▪^,^35^]. The addition of neuroprotective substances such as memantine and donepezil, an NMDA-receptor antagonist frequently applied in the treatment of Alzheimer's disease, might aid to improve patient outcomes. While NCCN guidelines consider memantine, which has been investigated for WBRT, but not for PCI [9,38,43]. These substances were linked to reduced decline and slight enhancement of cognitive functions in two phase III trials, although the results did not reach statistical significance [44,45]. In addition to memantine, the use of lithium (NCT01553916) and donepezil (NCT00006349) for similar protective roles in PCI treatment have been investigated.

The beneficial role of PCI, especially in light of immunotherapy administration, remains controversial. Results emerging from a phase III Japanese trial further questioned its legitimacy by proving that PCI did not lead to longer OS compared with active MRI surveillance in patients with ES-SCLC, although administration of PCI in ES has never been a standard treatment. According to this study, PCI should not be administered ES-patients who have responded to initial CHT and have a confirmed absence of brain metastases, provided MRI surveillance is implemented [46]. Other ongoing studies such as MAVERICK, PRIMAlung, NCT05651802, or NCT04829708 aim to discover whether MRI surveillance will be able to completely replace PCI in future SCLC management protocols. (Table 1).

## SUBTYPE-SPECIFIC VARIABILITY IN RESPONSE TO THERAPY

Even though both preclinical and clinical studies have offered promising results in recent years, significant changes in SCLC treatment have not transpired [47]. This is partly because of intratumoral heterogeneity, the scarcity of surgically resected tissue samples for research purposes, or the lack of potentially targetable driver mutations [1^▪▪^,^48^]. In addition, most clinical trials are still conducted on non-selected patient populations irrespective of potential molecular subtypes [47].

**FIGURE 1:**
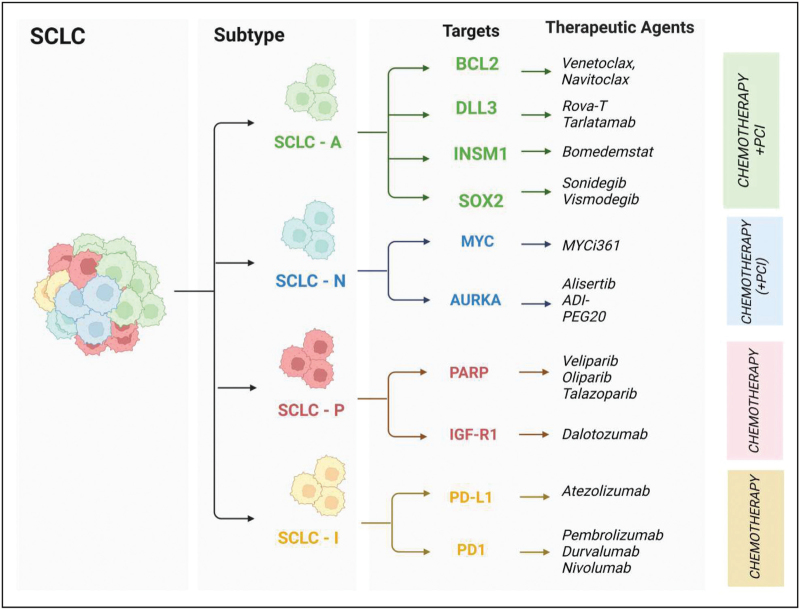
Potential and already implemented therapeutic agent-based subtype specific targets in small cell lung cancer.

Focusing on biological and clinicopathological differences between SCLC subtypes in the search for potential therapeutic targets has been an emerging interest in recent years (Fig. 1). The next paragraphs give a short summary of specific therapeutic vulnerabilities among molecular subgroups.

As for SCLC-A, higher expression levels of the antiapoptotic protein BCL-2 have been observed. We recently demonstrated that BCL-2 inhibitors (e.g. venetoclax) represent a potential therapeutic agent for this subgroup [47]. Furthermore, the ASCL1-dominant subtype has been associated with a transcriptional interaction with DLL3 in cells where the Notch pathway is downregulated, constituting a potential subtype-specific susceptibility to DLL3 inhibitors [49,50]. The FDA recently approved the bispecific T-cell engager tarlatamab for recurrent SCLC. This agent selectively targets DLL3 on tumor cells and CD3 on T cells [51^▪▪^]. Although tarlatamab is currently administered to all SCLC patients regardless of their molecular landscape, its application in a subtype-specific manner might further increase the therapeutic efficacy in the future. SCLC-A is also characterized by high levels of the NE transcription factor INSM1 [52^▪▪^]. LSD1 inhibitors prevent the expression of ASCL1 by disrupting the interactions between LSD1 and INSM1 [53]. In addition, given the high expression of the SOX2 oncogene, hedgehog signal cascade inhibitors may present a new possibility for SCLC-A [53]. As the tumor suppressor gene *CREBBP* is downregulated in ASCL1-expressing SCLCs, histone deacetylase inhibitors could potentially be effective [54]. Lastly, studies have shown that SCLC-A is more chemo-sensitive and radiosensitive than NE-low phenotypes. PCI and CRT might therefore be more suitable treatments in this particular subset of SCLC [55^▪^,^56^].

NEUROD1-driven tumors (SCLC-N) have been shown to have MYC oncogene amplification and lower NE profiles, making MYC inhibitors a possible therapeutic option [57]. SCLC-N has also been proven to exhibit higher AURKA activity and increased arginine biosynthesis. Consequently, AURKA inhibition or pegylated arginine deaminase are likely effective treatment strategies in this subtype [1^▪▪^,^58^].

SCLC-P tumors display a non-NE phenotype. This subgroup is suspected to be the most sensitive to PARP inhibitors and nucleoside analogue therapy [1^▪▪^,^59^]. Recent studies suggest that IGF-R1 inhibition could represent a novel therapeutic approach in the POU2F3-driven subtype [1^▪▪^,^59^].

Lastly, SCLC-I is classified by an inflamed phenotype with immune oasis characteristics and high immune-checkpoint marker expression [1^▪▪^]. Inhibitors of the PD-1/PD-L1 axis have proven to be effective and have been included in treatment protocols in ES settings [60]. Results from the retrospective analysis of IMpower133 data suggest that patients with SCLC-I features benefitted more from immune-checkpoint inhibitor treatment [61^▪^]. Moreover, SCLC-I has a presumably high YAP1 expression, which exhibits vulnerability to mTOR, PLK, and CDK4/6 inhibition [10].

## CONCLUSION

SCLC is one of the most aggressive malignant diseases. Despite the introduction of immunotherapy to treatment protocols for ES patients, there have been no significant changes in therapeutic approaches in the last decades. The objective of this review was to shed light on the role of PCI in SCLC in the era of immunotherapy. We also aimed to highlight potential next steps in SCLC treatment strategies while keeping molecular subtyping efforts in mind.

Several trial results have demonstrated the use of PCI in decreasing the occurrence of brain metastases and also prolonging OS in SCLC. PCI is routinely applied in LS-SCLC, with good responses to CRT, whereas the benefit at ES from PCI treatment is limited. Although serious PCI-related side effects have led to HA-PCI application and co-administration of memantine, these measures should be carefully considered before use. Introduction of immunotherapy might further limit the administration of PCI in favor of MRI-surveillance followed by stereotactic radiotherapy or radiosurgery.

Considering the improved quality of brain imaging techniques and the potentially better control of microscopic intracranial seeding provided by immunotherapy (even after the termination of CRT administration as per therapeutic guidelines), we believe, that the omission of PCI can be discussed for patients in favor of active MRI surveillance complemented with immunotherapy. This especially holds true for patients of the SCLC-I subtype with encouraging responses to immunotherapy. While immunotherapy may be less effective in patients with SCLC-A profiles, this subgroup likely profits most from RT, especially when brain metastases appear. PCI administration is therefore likely beneficial.

In summary, PCI remains the recommended standard-of-care for patients with good response after RCT for LS-SCLC and in good general condition, but the therapeutic value of PCI in SCLC is increasingly challenged, with ongoing trials investigating the possibility of replacement through MRI surveillance. Future SCLC studies in the era of immunotherapy are required to help select the patient population that profits more from PCI than MRI surveillance alone, thereby improving patient outcomes in this recalcitrant disease.

## Acknowledgements

*None.*

### Financial support and sponsorship

*B.D. was supported by the Austrian Science Fund (FWF I3522, FWF I3977, and I4677) and the ‘BIOSMALL’ EU HORIZON-MSCA-2022-SE-01 project. B.D. and Z.M. were supported by funding from the Hungarian National Research, Development, and Innovation Office (2020-1.1.6-JÖVŐ, TKP2021-EGA-33, FK-143751 and FK-147045). Z.M. was supported by the New National Excellence Program of the Ministry for Innovation and Technology of Hungary (UNKP-20-3, UNKP-21-3 and UNKP-23-5), and by the Bolyai Research Scholarship of the Hungarian Academy of Sciences. Z.M. is also the recipient of the International Association for the Study of Lung Cancer/International Lung Cancer Foundation Young Investigator Grant (2022). V.P. was supported by the New National Excellence Program of the Ministry for Innovation and Technology of Hungary (UNKP-23-3).*

### Conflicts of interest

*There are no conflicts of interest.*

## REFERENCES AND RECOMMENDED READING

Papers of particular interest, published within the annual period of review, have been highlighted as:

▪ of special interest
▪▪ of outstanding interest
